# PKC-δ deficiency in B cells displays osteopenia accompanied with upregulation of RANKL expression and osteoclast–osteoblast uncoupling

**DOI:** 10.1038/s41419-020-02947-3

**Published:** 2020-09-16

**Authors:** Shangfu Li, Qiuli Liu, Depeng Wu, Tianwei He, Jinbo Yuan, Heng Qiu, Jennifer Tickner, Song Guo Zheng, Xiaojuan Li, Jiake Xu, Limin Rong

**Affiliations:** 1 Department of Spine Surgery, The Third Affiliated Hospital of Sun Yat-sen University, Guangzhou Guangdong, China; 2 Guangdong Provincial Center for Quality Control of Minimally Invasive Spine Surgery, Guangzhou, China; 3 Guangdong Provincial Center for Engineering and Technology Research of Minimally Invasive Spine Surgery, Guangzhou, China; 4 The Biotherapy Center, The Third Affiliated Hospital of Sun Yat-sen University, Guangzhou Guangdong, China; 5 School of Biomedical Sciences, The University of Western Australia, Perth, WA Australia; 6 Department of Internal Medicine, The Ohio State University Wexner Medical Center, Columbus, OH USA; 7 Laboratory of Anti-inflammatory and Immunomodulatory Pharmacology, Southern Medical University, Guangzhou Guangdong, China

**Keywords:** Mechanisms of disease, Autoimmune diseases

## Abstract

PKC-δ is an important molecule for B-cell proliferation and tolerance. B cells have long been recognized to play a part in osteoimmunology and pathological bone loss. However, the role of B cells with PKC-δ deficiency in bone homeostasis and the underlying mechanisms are unknown. We generated mice with PKC-δ deletion selectively in B cells by crossing PKC-δ-loxP mice with CD19-Cre mice. We studied their bone phenotype using micro-CT and histology. Next, immune organs were obtained and analyzed. Western blotting was used to determine the RANKL/OPG ratio in vitro in B-cell cultures, ELISA assay and immunohistochemistry were used to analyze in vivo RANKL/OPG balance in serum and bone sections respectively. Finally, we utilized osteoclastogenesis to study osteoclast function via hydroxyapatite resorption assay, and isolated primary calvaria osteoblasts to investigate osteoblast proliferation and differentiation. We also investigated osteoclast and osteoblast biology in co-culture with B-cell supernatants. We found that mice with PKC-δ deficiency in B cells displayed an osteopenia phenotype in the trabecular and cortical compartment of long bones. In addition, PKC-δ deletion resulted in changes of trabecular bone structure in association with activation of osteoclast bone resorption and decrease in osteoblast parameters. As expected, inactivation of PKC-δ in B cells resulted in changes in spleen B-cell number, function, and distribution. Consistently, the RANKL/OPG ratio was elevated remarkably in B-cell culture, in the serum and in bone specimens after loss of PKC-δ in B cells. Finally, in vitro analysis revealed that PKC-δ ablation suppressed osteoclast differentiation and function but co-culture with B-cell supernatant reversed the suppression effect, as well as impaired osteoblast proliferation and function, indicative of osteoclast–osteoblast uncoupling. In conclusion, PKC-δ plays an important role in the interplay between B cells in the immune system and bone cells in the pathogenesis of bone lytic diseases.

## Introduction

Osteoimmunology was an interdisciplinary subject coined by Arron in 2000 to highlight the reciprocal interactions between the skeletal and immune systems^1^. Given the tight anatomical and physiological coexistence of B cells and the bone-forming units in the bone marrow, accumulating evidence indicated an important role for B cells in osteoimmunological regulation^2^. Recent findings indicating that B cells are active regulators of the receptor activator of NF-κB (RANK)/receptor activator of NF-κB ligand (RANKL)/osteoprotegerin (OPG) axis^3–5^, and that RANKL expression by B cells contributed to ovariectomy-induced bone loss further support this assumption^6^.

Protein kinase C δ (PKC-δ) was first cloned over 30 years ago^7^ and it is notably identified as an essential regulator of peripheral B-cell development and a critical regulator of immune homeostasis^8^. In 2002, PKC-δ null mice, which develop systemic autoimmunity revealed an essential role for this kinase in B-cell homeostasis and tolerance^9,10^. In humans, B cells are considered central in lupus pathogenesis and PKC-δ deficiency was identified as the first B-cell-related subset of monogenic lupus. Biallelic mutations in *PRKCD* (the gene that encodes PKC-δ) are associated with lupus and lymphoproliferative diseases because PKC-δ displays proapoptotic activity and is crucial to eliminate self-reactive transitional B cells^11–14^. These findings further confirmed PKC-δ as a critical proapopotic molecule essential in B-cell survival and apoptosis.

Bone cells (such as osteoclasts (OCs), osteoblasts (OBs), and osteocytes) and hematopoietic cells share the same microenvironment in the bone marrow and interact with each other to cooperatively regulate the functional activities of the bone system. PKC-δ deficiency perturbs bone homeostasis by selective uncoupling of Cathepsin K (CTSK) secretion and ruffled border formation in OCs^15^, and loss of PKC-δ protected against LPS-induced osteolysis owing to an intrinsic defect in osteoclastic bone resorption^16^. In addition, PKC modulated the synthesis of nitric oxide by OBs^17^ and noncanonical Wnt signaling through G-protein-linked PKC-δ activation promoted bone formation^18^. Moreover, PKC-δ played an important role in the osteochondral plasticity of the interface between articular cartilage and the osteochondral junction^19^. These studies revealed that PKC-δ not only played an essential role in immunity but also in skeletal biology. RANKL interacts with two receptors, one functionally called RANK and the other a decoy named OPG. RANKL is a key OC differentiation factor and was found to play an essential role not only in the development of immune organs and bones, but also in autoimmune diseases affecting bone^20^. In addition, B-lymphoid lineage cells are a major source of endogenous RANKL in bone marrow and support OC differentiation in vitro^21^. However, the association between PKC-δ function and RANKL expression in B cells, and its role in bone homeostasis remain unclear.

Our study aimed to investigate the important role of PKC-δ in B cells and its subsequent effects on OC and OB biology by using a Cre-loxP-based conditional knockout (cKO) technology to selectively inactivate PKC-δ in B cells, which could help to shed more light on our understanding of osteoimmunology-related disease, such as rheumatoid arthritis and osteoporosis.

## Results

### PKC-δ conditional knockout in B cells results in osteopenia and altered bone microstructure in mice

Firstly, we established and confirmed CD19-driven PKC-δ deletion in B cells in mice. We used the conditional PKC-δ allele in which exon 7 is flanked by loxP sites. Cre-mediated deletion of exon 7 results in a PKC-δ null allele in B cells (Supplementary Fig. 1a). Efficiency of Cre-mediated deletion of PKC-δ exon 7 and consequent loss of PKC-δ expression in B cells was confirmed by DNA PCR for the deleted and floxed alleles (Supplementary Fig. 1b). Further, significant decrease of PKC-δ mRNA (Supplementary Fig. 1c) and almost absence of protein expression (Supplementary Fig. 1d) in B cells were verified.

**Fig. 1:**
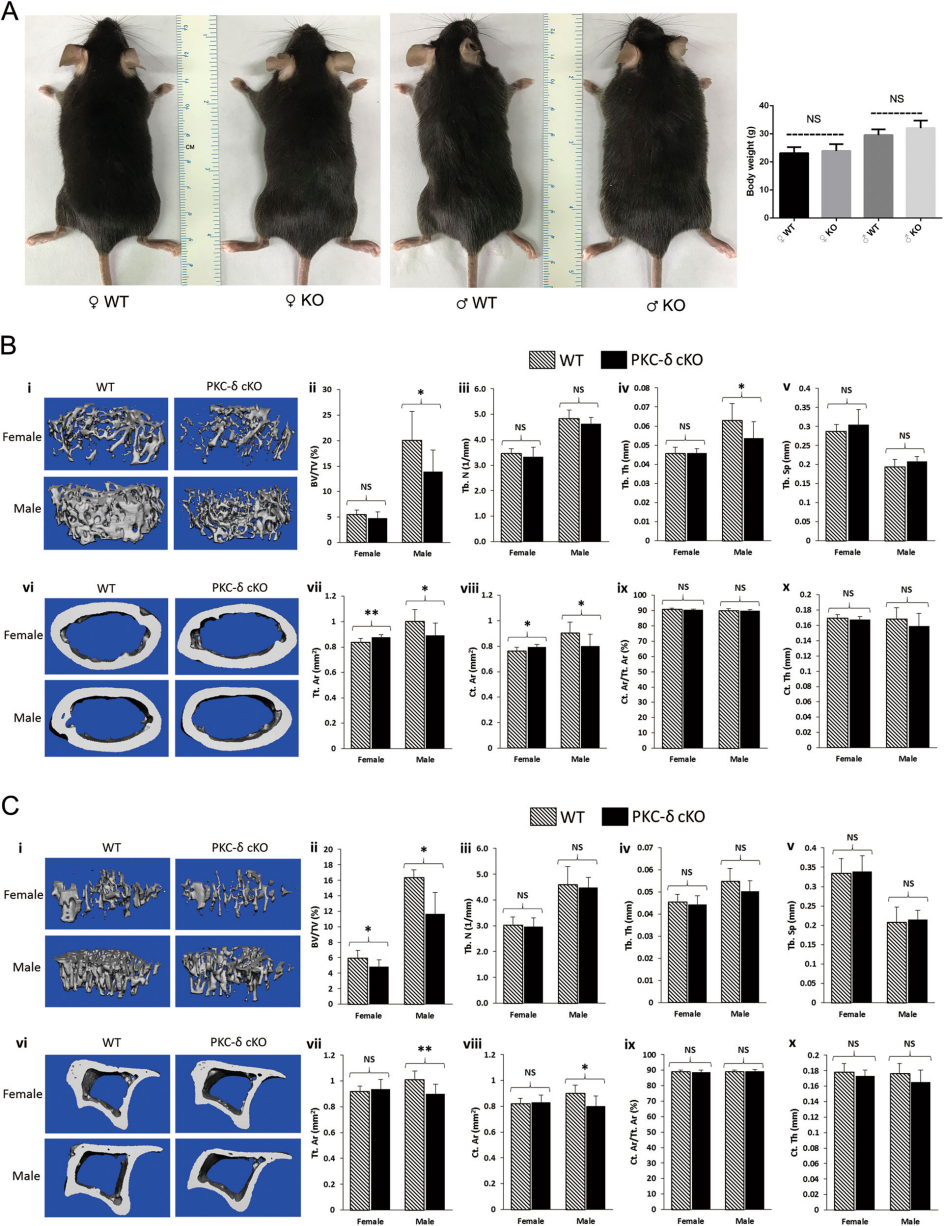
Micro-CT analysis of hind limbs revealing an osteoporotic phenotype in 12-week-old mice with PKC-δ conditional knockout in B cells. **a** Representative images and body weight of the mice at 12 weeks of age (WT wild-type, KO knockout). NS = non-significant compared with WT controls; **b,** **c** Representative 3D reconstructions of trabecular and cortical bone and bone parameters assessed by micro-CT in distal femur (**b** i–x) and proximal tibia (**c** i–x) in age- and sex-matched WT and PKC-δ conditional knockout (cKO) mice, respectively (male WT *n* = 6, male cKO *n* = 7, female WT *n* = 7, female cKO *n* = 7). Trabecular bone parameters (**b** ii–v and **c** ii–v) are shown as trabecular bone volume fraction (BV/TV, %; **b** ii and **c** ii), trabecular number (Tb.N, 1/mm; **b** iii and **c** iii), trabecular thickness (Tb.Th, mm; **b** iv and **c** iv) and trabecular separation (Tb.Sp, mm; **b** v and **c** v). Micro-CT analysis of cortical bone parameters (**b** vii–x and **c** vii–x) are shown as total cortical area (Tt.Ar, mm^2^; **b** vii and **c** vii), cortical bone area (Ct.Ar, mm^2^; **b** viii and **c** viii), cortical area fraction (Ct.Ar/Tt.Ar, %; **b** ix and **c** ix) and cortical thickness (Ct.Th, μm; **b** x and **c** x). Data are presented as mean ± SD. *p < 0.05, **p < 0.01, NS = non-significant compared with WT controls.

To determine the contribution of PKC-δ cKO in B cells in skeletal development and bone homeostasis, we firstly analyzed the gross appearance of 3-month-old PKC-δ cKO mice. Interestingly, there were no significant changes in both male and female mice regarding their body weight (Fig. 1a), suggesting cKO mice had a similar body structure to that of WT littermates. We further examined the bone microstructure using microcomputed tomography (micro-CT). Interestingly, micro-CT analysis revealed that PKC-δ deficiency changed bone volume and microstructure in both femur (Fig. 1b) and tibia (Fig. 1c) in cKO mice compared to age- and sex-matched WT littermates. In the femur, the percentage of trabecular bone volume versus total volume (BV/TV, Fig. 1bii) and trabecular thickness (Tb.Th, Fig. 1biv) were all significantly reduced in male mice but not in female mice in the trabecular bone. There were a trend of decrease in trabecular number (Tb.N, Fig. 1biii) and a trend of increase in trabecular separation (Tb.Sp, Fig. 1bv); however, no significant statistical differences were found. In the femoral cortical bone, total cortical area (Tt.Ar, Fig. 1bvii) and cortical bone area (Ct.Ar, Fig. 1bviii) were also all significantly reduced in male mice but increased for female mice compared with the WT controls. However, there were no significant changes in cortical area fraction (Ct.Ar/Tt.Ar, Fig. 1bix) and cortical thickness (Ct.Th, Fig. 1bx). In the tibia, for the trabecular bone micro-CT parameters, only BV/TV (Fig. 1cii) was significantly reduced for both male and female mice compared with WT control mice. No significant differences were found in Tb.N (Fig. 1ciii), Tb.Th (Fig. 1civ) and Tb.Sp (Fig. 1cv). For the tibial cortical bone parameters, there were significant decreases in male but not in female mice regarding Tt.Ar (Fig. 1cvii) and Ct.Ar (Fig. 1cviii). However, no significant differences were found in Ct.Ar/ Tt.Ar (Fig. 1cix) and Ct.Th (Fig. 1cx).

Taken together, these findings show there is an osteopenic phenotype in both trabecular bone and cortical bone after PKC-δ ablation in B cells.

### Decreased bone fraction and osteoblasts and increased osteoclasts in trabecular bone of PKC-δ conditional knockout mice

**Fig. 2:**
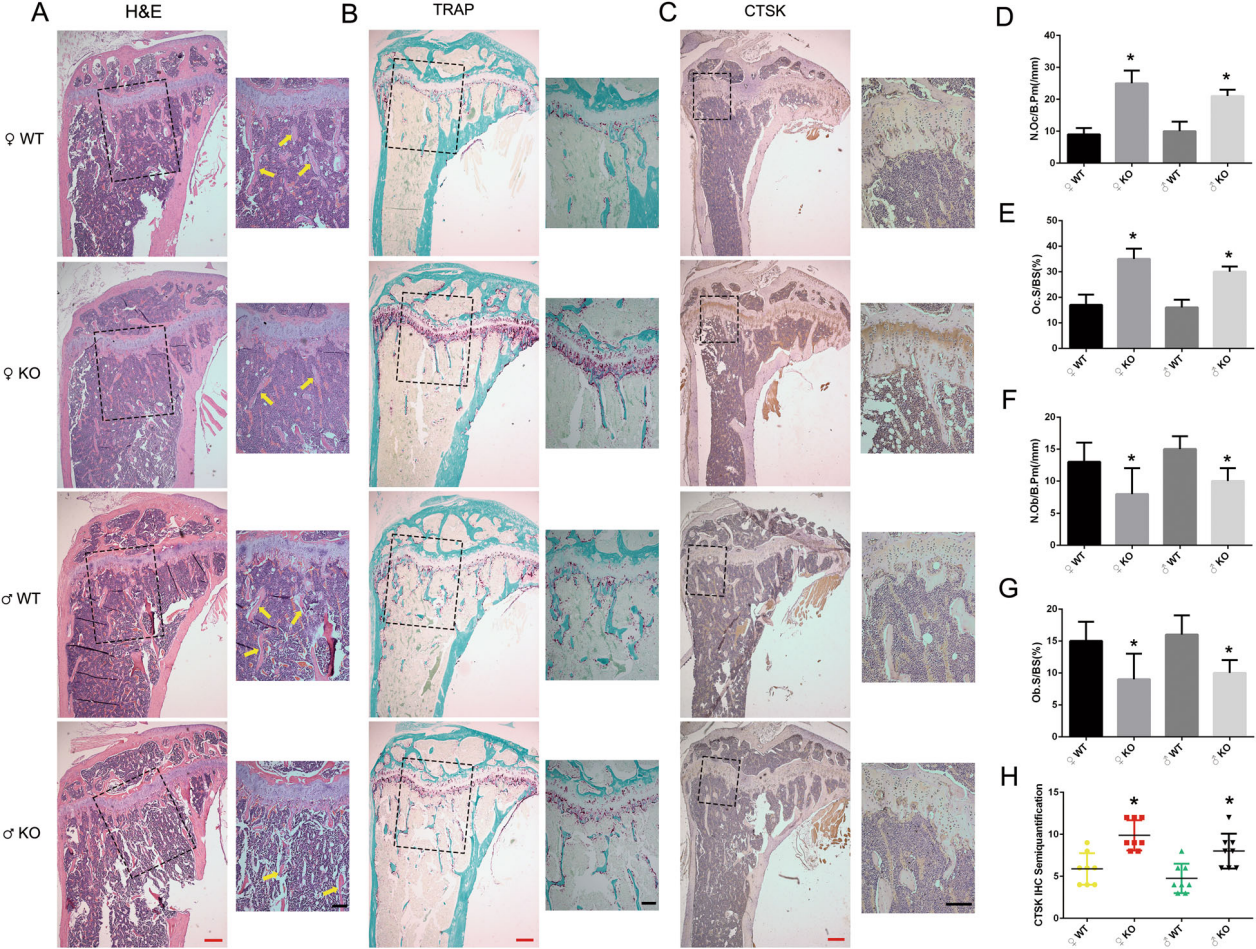
Histological analysis of wide type and PKC-δ conditional knockout proximal tibias from 3-month-old mice. **a**–**c** Representative low-power images of H&E/TRAP/CTSK stained tibia sections. Higher magnification micrograph of area within square in **a**–**c** was presented at the right side of each, and yellow arrows in **a** indicate trabecular bone within the tibia. Red bar and black bar represent 200 μm and 100 μm, respectively. **d**–**g** Quantitative histomorphometric analysis of bone parameters: **d** Number of osteoclasts per bone perimeter (N.Oc/B.Pm, mm^−1^), **e** Osteoclast surface relative to bone surface (Oc.S/BS, %), **f** Number of osteoblasts per bone perimeter (N.Ob/ B.Pm, mm^−1^), **g** Osteoblast surface relative to bone surface (Ob.S/BS, %). **h** Semi-quantification analysis of CTSK IHC staining. Male WT *n* = 6, male cKO *n* = 7, female WT *n* = 7, female cKO *n* = 7. Bar charts represent mean ± SD. *p < 0.05 compared with WT controls.

To gain further insight into the in vivo osteopenic phenotype of the cKO mice, bone histomorphometry was performed on decalcified sections stained with hematoxylin and eosin (H&E) (Fig. 2a) and for tartrate-resistant acid phosphatase (TRAP) activity (Fig. 2b). Consistent with the bone loss phenotype demonstrated by micro-CT, H&E staining of tibia from 3-month-old cKO mice showed that trabecular bone was greatly reduced when compared to WT mice (Fig. 2a). Bone remodeling is a highly coordinated process requiring bone resorption by OC and bone formation by OB and is necessary to maintain mineral homeostasis^22^. We thus further investigated OB and OC number and distribution in vivo in bone sections to elucidate the osteopenic phenotype. Consistent with the micro-CT findings, analysis of OC parameters using TRAP stained sections (Fig. 2b) revealed that cKO mice exhibited a significant increase in the number of OCs per bone perimeter (Fig. 2d) and OC surface per bone surface (Fig. 2e). We further verified our finding by examining the expression of CTSK, an important biomarker for OC function, using immunohistochemistry (IHC) staining. As expected, semi-quantitative analysis showed increased CTSK activity after inactivation of PKC-δ in B cells when compared with the WT controls (Fig. 2h). For the in vivo OB parameters, as expected, H&E staining revealed that OB number per bone perimeter (Fig. 2f) and OB surface per bone surface (Fig. 2g) in the tibia displayed significant decrease when compared with the WT littermates. Osteocalcin IHC staining further verified the reduction of osteoblasts in the trabecular bone in mice with B-cell-specific deletion of PKC-δ (Supplementary Fig. 2).

Articular cartilage plays a critical role in bone and cartilage homeostasis^23^, previous study showed that PKC-δ is an important regulator of osteochondral plasticity^19^. Next, we examined cartilage changes using Safranin O Fast Green Staining, no significant differences regarding articular cartilage thickness and no osteoarthritis-like features were found as shown for the representative images (Supplementary Fig. 3a). Bone is a dynamic organ composed of organic and inorganic elements^24^. Representative images of von Kossa staining showed decreased inorganic contents in the trabecular bone of PKC-δ cKO mice (Supplementary Fig. 3c). No changes to organic components were observed as shown in the representative images of Masson’s trichrome staining (Supplementary Fig. 3b).

Collectively, these results suggest that the osteopenic phenotype after cKO of PKC-δ in B cells was mainly due to OC and OB changes.

### PKC-δ deficiency in B cells was accompanied by changes in B-cell number, function, and distribution

**Fig. 3:**
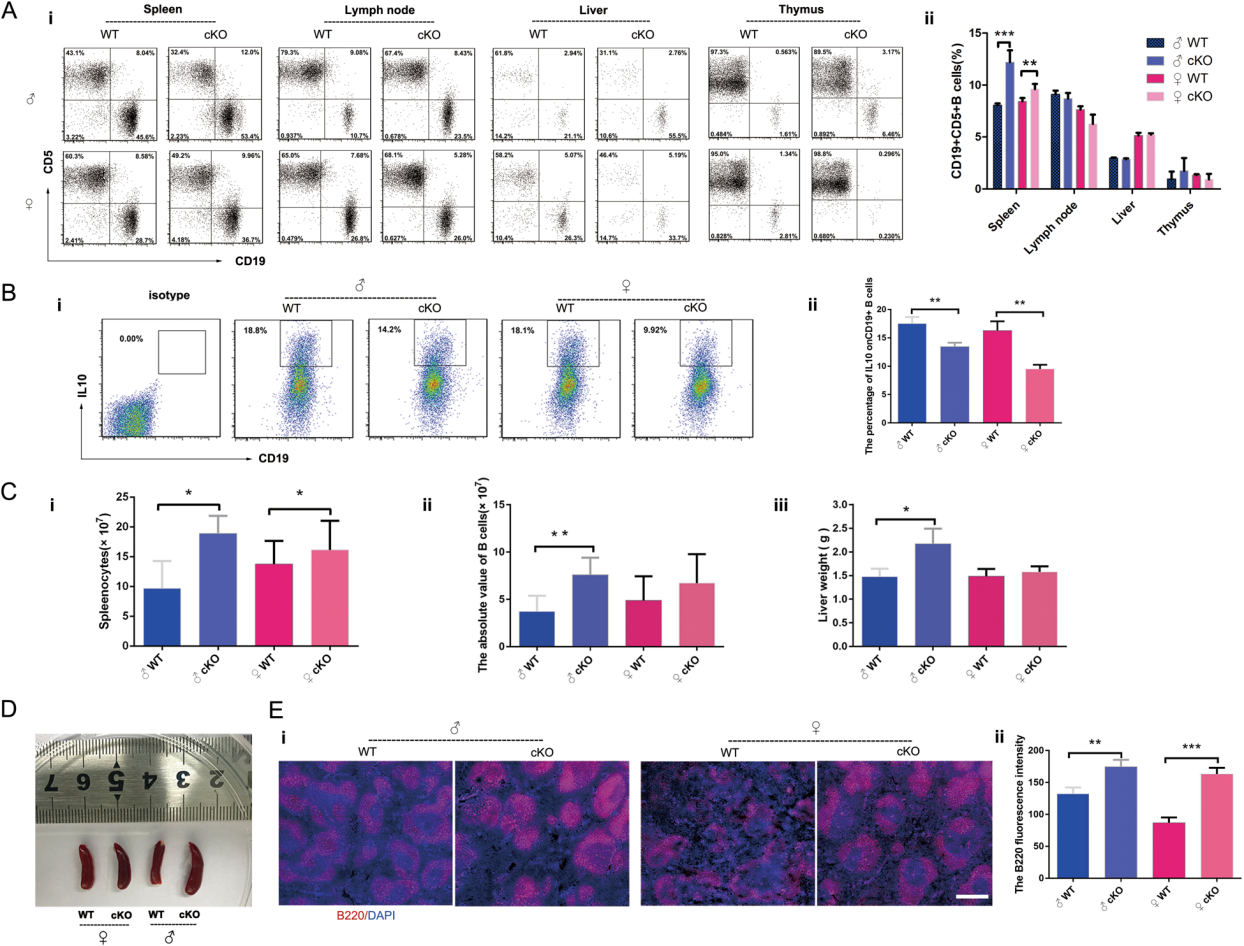
PKC-δ deficiency in B cells is accompanied by changes in B-cell number, function, and distribution. **a** Flow-cytometric analysis of the percentage of CD19^+^CD5^+^ B cells in spleen, lymph nodes, liver and thymus from WT and PKC-δ cKO mice (i), with bar charts showing the quantification (ii); **b** Flow-cytometric analysis of the percentage of IL-10^+^CD19^+^ B cells in splenocytes from WT and PKC-δ cKO mice (i), with bar charts showing the quantification (ii); **c** The number of splenocytes was counted after single-cell suspensions were prepared from pooled spleens of WT and PKC-δ cKO mice (i). The absolute number of splenic CD19^+^ B cells was determined after B-cell sorting (ii). The liver weight was measured after complete separation of liver from WT and PKC-δ cKO mice (iii); **d** Representative image of general appearance of spleens from WT and PKC-δ cKO mice; **e** Representative images of immunofluorescent staining from lymphoid follicles in the spleen of WT and PKC-δ cKO mice (i). Cryosections were stained with anti-B220 (Red) and DNA was stained with DAPI (blue), bar represents 200 μm. B220 fluorescence intensity was measured using ImageJ software (ii). Data were presented as mean ± SD (*n* = 4). *p < 0.05, **p < 0.01, ***p < 0.001 compared with WT control.

PKC-δ plays an important role in immune organ development and skeletal diseases^2,8^. We further investigated its role in B-cell development and function. It has been reported that PKC-δ null mice had an increase of B-cell proliferation^10^, B1a cells have been implicated in the development of autoimmune arthritis through RANKL-mediated osteoclastogenesis^25^, but it is not clear whether PKC-δ deficiency selectively in B cells will affect B1a cells. Hence, we systematically compared the percentage of B1a cells (mainly characterized by expression both of CD19 and CD5) in different immune organs, and investigated changes in the number, distribution, and function of B cells after PKC-δ depletion. We obtained single-cell suspensions from spleen, lymph nodes, liver, and thymus, then analyzed the percentage of B1a cells by flow cytometry (Fig. 3ai). The step by step gating schedule is shown in Supplementary Fig. 4. Interestingly, we found that the percentage of B1a cells was significantly increased only in spleen after PKC-δ ablation, especially in male mice (Fig. 3aii). We further evaluated the change of B1a cells function by detecting the important anti-inflammatory factor IL-10 (Fig. 3bi), we found that their ability to secrete IL-10 was significantly reduced in B cells with depletion of PKC-δ (Fig. 3bii). Next, we separately evaluated the total number of spleen cells in PKC-δ-deficient mice, and the number of purified B cells in the spleen. As shown in Fig. 3c, the number of total spleen cells was increased in both female and male mice after PKC-δ inactivation (Fig. 3ci), while the number of purified B cells obtained from the total spleen cells (the absolute value) was significantly increased only in male mice but not in female mice after ablating PKC-δ (Fig. 3cii). Interestingly, an increased liver weight (Fig. 3ciii) and larger spleen (Fig. 3d) were also observed in male mice but not in female mice after PKC-δ cKO. Consistently, we also found that the B cells were significantly increased (Fig. 3ei, indicated by red color) after PKC-δ delection by measuring the fluorescence intensity of B220 (Fig. 3eii), which was expressed by the B-cell lineage from early pro-B cells to mature B cells. Intriguingly, we observed that the B-cell area in the spleen became larger both in male and female mice after PKC-δ cKO (Fig. 3ei).

Taken together, our data showed that PKC-δ deficiency in B cells was accompanied by changes in B-cell number, function, and distribution mainly in the spleen.

### PKC-δ deficiency in B cells increased RANKL/OPG ratio

**Fig. 4:**
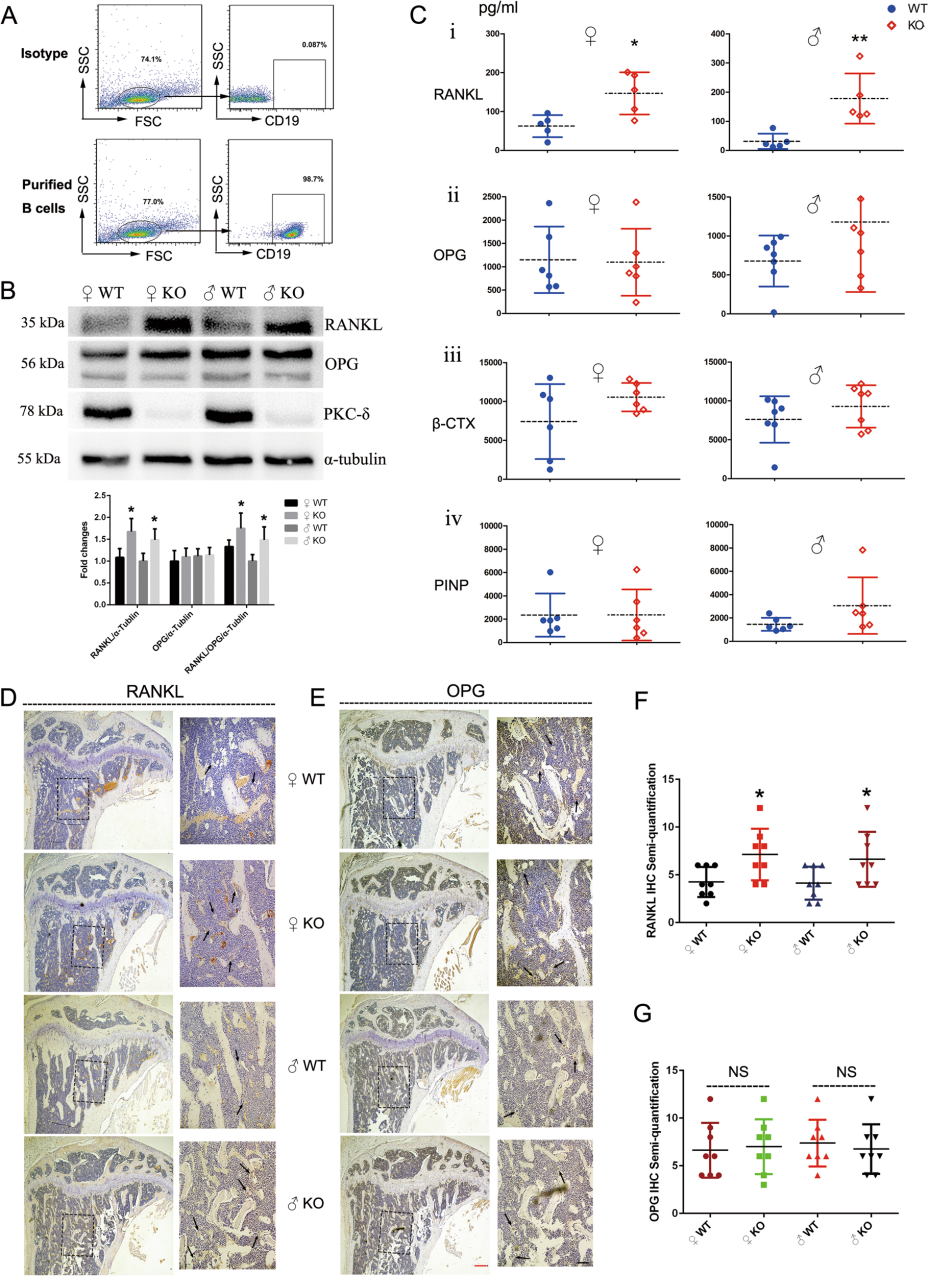
PKC-δ deficiency in B cells elevated RANKL/OPG ratio in both cell culture and serum and increased RANKL expression in the trabecular bone. **a** Flow-cytometry plots representing the purity of B cells after sorting; **b** Expression of PKC-δ, OPG, and RANKL in B cells from WT and PKC-δ cKO mice was detected by western blotting, with semi-quantitative analysis; **c** Blood was obtained from the fundus vein of WT and PKC-δ cKO mice. The concentrations of RANKL, OPG, β-CTX, and PINP were determined by ELISA in the serum. Each data point was from an individual mouse and means were indicated by dashed horizontal lines. **d**, **e** Representative images of RANKL (**d**) and OPG (**e**) immunohistochemistry stained tibia sections of 3-month-old PKC-δ cKO and age-sex-matched wild-type mice. Higher magnification micrograph of area within square in **d**, **e** is presented at the right side of each. Arrows indicate the positive staining of osteoclasts in the trabecular bone within the tibia. Red bar and black bar represent 200 μm and 50 μm, respectively. **f**, **g** Semi-quantitative analysis of RANKL (**f**) and OPG (**g**) immunohistochemistry staining. Bar charts represent mean ± SD. *p < 0.05, **p < 0.01, NS = non-significant compared with WT control group.

RANKL/OPG/RANK signaling regulates numerous physiological processes including bone remodeling and lymph node organogenesis^20^. RANKL is best known for its indispensable role in promoting differentiation of mature OCs^26^. We thus investigated the RANKL/OPG ratio after ablation of PKC-δ in B cells. Firstly, we assessed the purity of B cells sorted by flow cytometry (98.7%, Fig. 4a). Next, we performed in vitro spleen-derived B-cell culture. Importantly, we found that the protein expression of RANKL and the ratio of RANKL/OPG were all significantly increased in female and male PKC-δ cKO mice (Fig. 4b). However, OPG protein expression was almost unchanged after ablation of PKC-δ, indicating that PKC-δ deficiency in B cells raised the RANKL/OPG ratio mainly due to the overexpression of RANKL. Furthermore, we collected the serum from 3-month-old mice to determine the concentration of OPG, RANKL and bone turnover markers of β-CTX and PINP by ELISA. As expected, the level of RANKL in PKC-δ cKO mice was remarkably increased both for male and female (Fig. 4ci), while there were no significant changes in OPG concentration after deletion of PKC-δ (Fig. 4cii). Unexpectedly, the bone resorption marker β-CTX (Fig. 4ciii) and the bone formation marker PINP (Fig. 4civ) were not significantly changed in both female and male mice after PKC-δ cKO. We next asked whether the elevation of RANKL both in B cells and in the serum would indirectly affect bone tissue, we thus performed RANKL and OPG IHC in tibia sections to verify our hypothesis. As showed in Fig. 4d–g, the staining intensity in bone cells, especially OCs, was significantly enhanced for RANKL IHC (Fig. 4d, f) in both female and male mice with ablation of PKC-δ in B cells. However, OPG staining intensity was unchanged as assessed by semi-quantitative analysis when compared with the WT littermates (Fig. 4e, g).

Accordingly, all these results indicated that PKC-δ deficiency in B cells led to increased RANKL/OPG ratio in cell cultures, in the serum and in the trabecular bone, and eventually caused an osteopenic phenotype.

### PKC-δ deletion in B cells resulted in osteoclast–osteoblast uncoupling

**Fig. 5:**
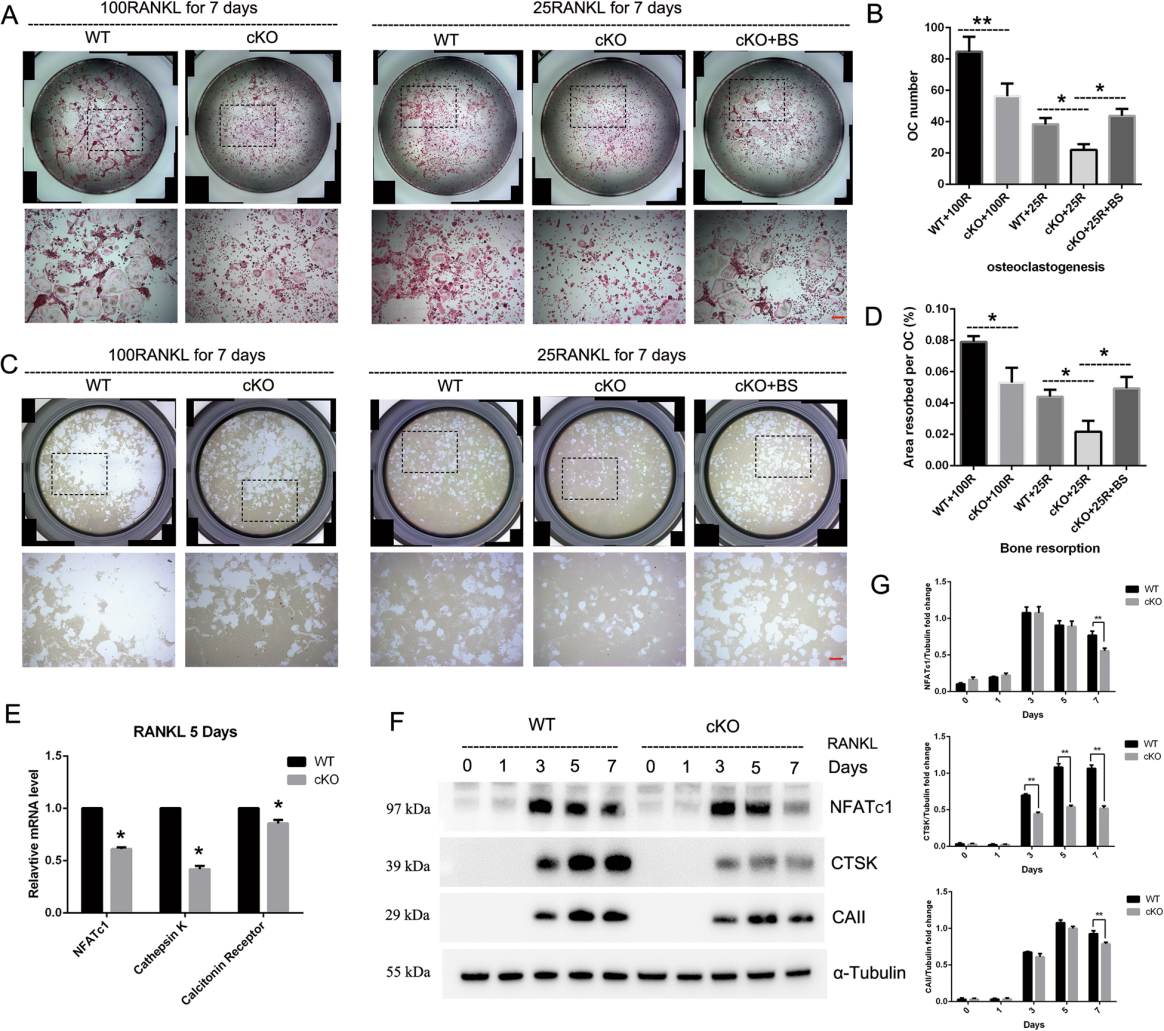
Deletion of PKC-δ selectively in B cells resulted in suppressing osteoclast differentiation and function, but B-cell supernatant co-culture exerted reversal effect on osteoclast formation and activation. **a** Representative images of osteoclasts with TRAP staining after 100 ng/ml or 25 ng/ml RANKL (with and without BS co-culture) induction for 7 days, the square in the upper images of each well indicate where the lower images were captured. Bar represents 200 μm; **b** Quantification of the number of osteoclasts, TRAP-positive cells containing three or more nuclei were counted as osteoclasts; **c** Representative images of eroded areas in hydroxyapatite-coated plates after 100 ng/ml or 25 ng/ml (with and without BS co-culture) RANKL stimulation for 5 days, the square in the upper images of each well indicate where the lower images were captured. Bar represents 200 μm; **d** Quantitative analysis of the resorbed proportion per osteoclast by measuring the area of the mineral coating removal; **e**–**g** Gene transcription (**e**) and protein expression **f**–**g** analysis of osteoclast-specific markers NFATc1, Cathepsin K (CTSK), Calcitonin Receptor and Carbonic Anhydrase II (CAII) by RT-PCR and western blotting. BS B-cell supernatant. All the experiments were carried out in triplicate from 12-week-old male WT and cKO mice, results are presented as mean ± SD. *p < 0.05, **p < 0.01 vs. WT control group.

In addition to the increased OCs observed in PKC-δ cKO mice in vivo, we further examined the effects of PKC-δ ablation in B cells on osteoclastogenesis and bone resorption in vitro. Surprisingly, BMMs from PKC-δ cKO produced significantly less OCs than WT controls after induction with 100 ng/ml RANKL for 7 days (Fig. 5a, b). We further assessed OC function by culturing mature osteoclasts derived from BMMs with 100 ng/ml RANKL for 7 days on hydroxyapatite-coated plates. Unexpectedly, the area of bone resorbed by PKC-δ cKO OCs was significantly decreased as compared to WT control (Fig. 5c, d). To confirm these findings, we detected the key genes and their downstream protein expression related to OC differentiation and function. As shown in Fig. 5e, the expression levels of these OC-related genes of *nfatc1*, *ctsk*, and *calcitonin receptor* were all remarkably decreased after stimulating with 100 ng/ml RANKL for 5 days in mice with deletion of PKC-δ. Moreover, the expression levels of downstream proteins related to OC bone resorption activity, such as NFATc1, CTSK, and Carbonic Anhydrase II (CAII), were also decreased at 7 days after 100 ng/ml RANKL stimulation in PKC-δ cKO mice (Fig. 5f, g). Because PKC-δ deficiency in B cells led to increased RANKL in both B-cell cultures and in the serum (Fig. 4a–c), we further performed B-cell co-culture experiments with OCs to verify our observations. B cells are prone to die in the culture medium, so we used B-cell supernatant (BS) for the co-culture experiments. As shown in Fig. 5a–d, osteoclastogenesis and bone resorption were all reduced after stimulation with 25 ng/ml RANKL for 7 days in PKC-δ cKO mice. However, as expected, BS co-culture significantly enhanced both OC formation and function and, surprisingly, reversed the suppression effect of PKC-δ deletion in B cells on OC biology.

**Fig. 6:**
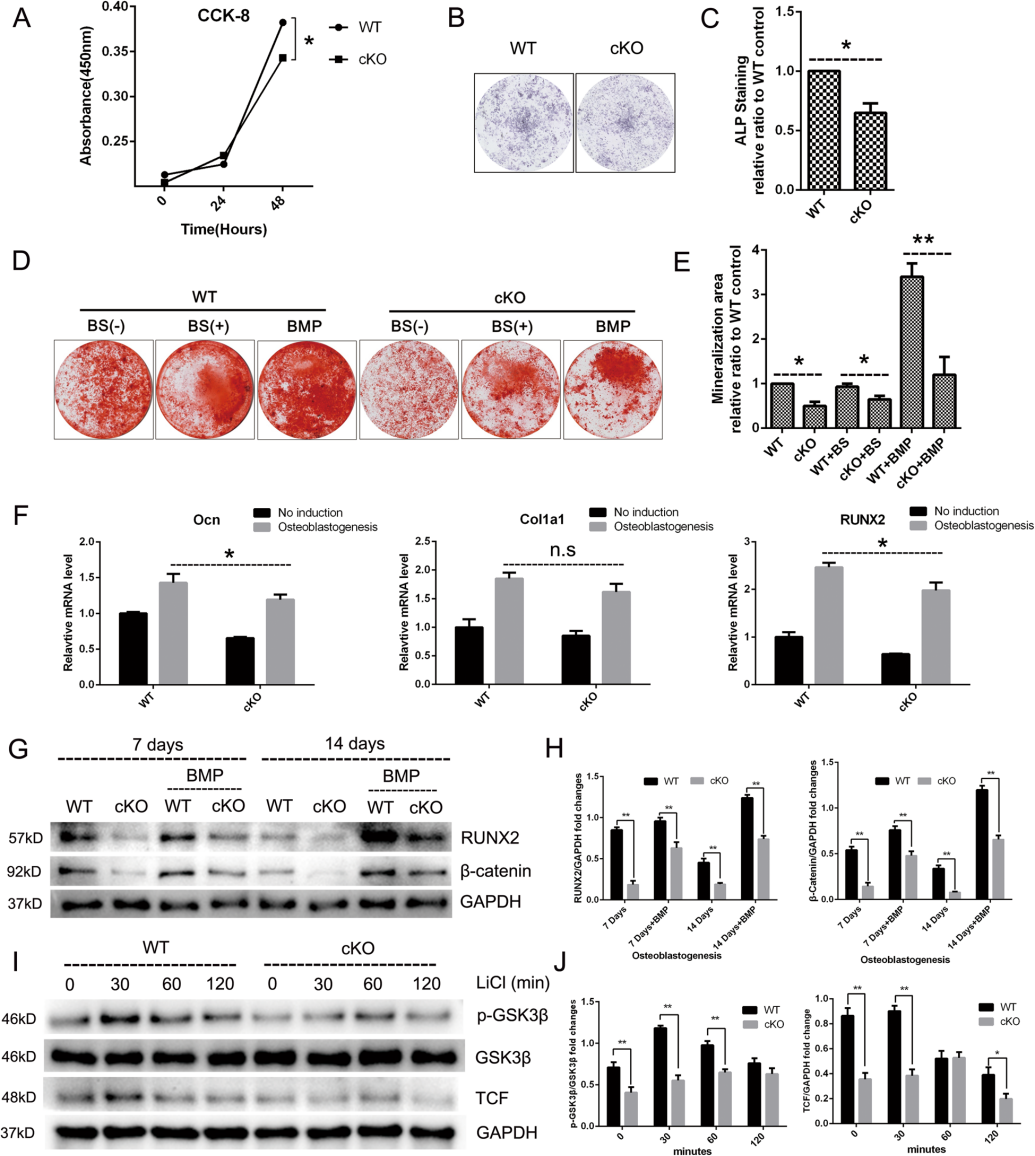
Deletion of PKC-δ selectively in B cells led to impairment of osteoblast proliferation, differentiation, and function. **a** Effect of PKC-δ cKO on osteoblast cell viability measured by the CCK-8 assay at day 1 and day 2. **b, c** Representative low-power images of alkaline phosphatase (ALP) staining (**b**) and quantitative analysis of ALP staining intensity relative to WT control group (**c**). **d**, **e** Representative low-power images showing the mineralized area stained with alizarin red (**d**) and quantitative analysis of the area of staining relative to WT control (**e**). **f** PKC-δ cKO suppressed osteoblast-specific genes transcription revealed by RT-PCR. *Runx2*, *Ocn*, and *Col1a1* gene transcription level were tested after 7 days of osteogenesis induction. **g**, **h** Representative images of western blotting reflecting the expression levels of β-catenin (Wnt/β-catenin pathway) and RUNX-2 normalized to GAPDH after 7 and 14 days of induction with and without BMP-2 co-culture (**g**), and quantitative analysis of the fold changes (**h**). BMP-2 was used as a positive control. **i,** **j** PKC-δ cKO interacted with GSK-3β phosphorylation and TCF transcription. Representative western blotting images of p-GSK-3β, GSK-3β, TCF, and GAPDH at 0, 30, 60, and 120 min stimulated with 20 mM LiCl (**i**) and quantitative analysis of the fold changes of p-GSK-3β and TCF expression (**j**). BS B-cell supernatant, BMP bone morphogenetic protein-2 (25 ng/ml). All the experiments were carried out in triplicate from 6-week-old male WT and cKO mice, results are presented as mean ± SD. n.s. no statistical significance, *p < 0.05, **p < 0.01 vs. WT control group.

Because OC–OB coupling plays the most important role during bone remodeling^22^, to investigate whether PKC-δ cKO has an indirect effect on OB biology, we evaluated the regulation of PKC-δ in OB proliferation and function. Proliferation rates of OBs derived from cKO mice were significantly reduced at 48 h (Fig. 6a). Furthermore, OBs derived from calvaria of cKO mice displayed a significant reduction of function in both a reduction in alkaline phosphatase (ALP) staining at days 7 (Fig. 6b, c) and in mineralization activity as evidenced by alizarin red staining at days 21, respectively. Moreover, the potent promotion effect of bone morphogenetic protein-2 (BMP-2) on osteoblastogenesis was greatly reduced after PKC-δ ablation as evidenced by alizarin red staining (Fig. 6d, e). We further carried out co-culture experiments with BS, which contained soluble RANKL during osteoblastogenesis. Surprisingly, BS co-culture had no effect on mineralized nodule formation (Fig. 6d, e). Furthermore, we did not observe any stimulation effect of recombinant (soluble) RANKL proteins (50 ng/ml and 100 ng/ml) on bone formation after treating MC3T3-E1 cells for 7 days (Supplementary Fig. 5). These results revealed that soluble RANKL, unlike membrane-bound RANKL^27^, had no effect on osteoblast formation and function. To further investigate the potential mechanisms of PKC-δ ablation in suppressing osteoblastogenesis at the molecular level, we firstly detected osteoblast-specific gene transcription and found that the relative mRNA expression of osteoblast transcription factors *ocn* and *runx2* were significantly downregulated by loss of PKC-δ, while *col1a1* had a trend of reduction without statistically significant difference (Fig. 6f). In addition, the protein expression level of osteoblast-specific RUNX2 was also reduced at day 7 and day 14 during osteoblastogenesis after loss of PKC-δ, moreover, the OB response to BMP-2 stimulation was markedly inhibited by PKC-δ ablation (Fig. 6g, h). To evaluate whether PKC-δ cKO suppresses the Wnt signaling pathway, we initially analyzed the expression levels of β-catenin during osteoblastogenesis and found it decreased at day 7 and day 14 in PKC-δ cKO mice (Fig. 6g, h). Given the fact that GSK-3β induced the phosphorylation and degradation of β-catenin in the absence of Wnt activation, we therefore investigated whether the impairment of the Wnt/β-catenin pathway by PKC-δ cKO is involved in GSK-3β activation. As shown in Fig. 6i–j, the phosphorylation of GSK-3β decreased when treated with LiCl at 0, 30, and 60 min after PKC-δ ablation. Moreover, the expression level of TCF, a key transcription factor of the Wnt/β-catenin pathway, was also significantly decreased after LiCl treatment. Collectively, PKC-δ ablation in B cells suppresses Wnt/β-catenin pathways accompanied by phosphorylation of GSK-3β.

In summary, PKC-δ ablation in B cells disturbs the OC–OB balance.

## Discussion

In this study, we found that mice with PKC-δ deficiency selectively in B cells displayed osteopenia and hyperproliferation of B cells. In addition, histological analysis showed that this is mainly due to increased OC number and CTSK activity and decreased OB parameters. More importantly, our work suggests that the RANKL/OPG ratio was increased both in vitro in B-cell culture as well as in vivo in serum and bone specimens. Furthermore, deletion of PKC-δ selectively in B cells disrupted OC–OB balance by suppressing OC differentiation and function but co-culture with B-cell supernatant reversed the suppression effect, as well as impairing OB proliferation and function. To the best of our knowledge, our data showed for the first time a close relationship between PKC-δ-deficient B cells in the immune system and bone cells in the bone microenvironment, indicating that PKC-δ may have an important role in the pathogenesis of osteoimmunology-related diseases.

Bone is a dynamic organ formed by deposition and resorption of the bone matrix, which are carried out by OBs and OCs respectively during bone homeostasis^22^. The most common disorder with this balancing act occurs when the rate of bone resorption exceeds accumulation, resulting in a loss of bone mass, such as is seen in osteoporosis, rheumatoid arthritis and cancer metastasis to bone^28^. It is interesting that changes in the number of B cells are related to osteoporosis. CD19^+^ B cells were low in osteoporotic women and positively correlated to bone mineral density^29^. However, B220^+^ B lymphocytes were selectively increased 2–4 weeks after ovariectomy and returned to normal when treated with estrogen^30^. Moreover, both estrogen deficiency^31^ and androgen shortage^32^ similarly stimulate B lymphopoiesis, which is involved in the mechanism of stimulated bone resorption. B cells are extensively linked to bone homeostasis from their development in the bone marrow to the homing of terminally differentiated plasma cells back to the bone marrow^2^. The bidirectional regulation of the skeletal system by B cells revolves the RANKL/OPG/RANK pathway^20^. Our work revealed a close relationship between B-cell RANKL expression and bone remodeling after PKC-δ ablation in B cells. RANKL is best known for its abundant expression in OBs and osteocytes^33,34^, it is also expressed in bone marrow and lymphoid tissues^35^. However, RANKL expression in immunocyte, including B cells and T cells, is much lower than that in OBs and osteocytes^36^. Resting B cells have not been shown to produce significant amounts of RANKL, but activated B cells serve as an important source of RANKL under inflammatory circumstances, such as rheumatoid arthritis^5^, periodontitis^37^, and HIV induced bone loss^38^. Our study showed that both B cells and RANKL expression were significantly increased in PKC-δ cKO mice, indicating that loss of PKC-δ had an activation effect on B-cell proliferation. It is interesting that B-cell-specific RANK-deficient mice did not exhibit any discernible osteopetrotic phenotype under physiological conditions^39^. However, RANKL expression by B lymphocytes contributed to ovariectomy-induced bone loss^6^, suggesting that B-cells-derived RANKL does contribute to the increase in OCs and cancellous bone loss that occurs in the pathological state of estrogen loss. All of our results supported the decreased bone mass observed in PKC-δ cKO mice except for the OC in vitro data, the discrepancy was also existed in PKC-δ null mice, which exhibited enhanced osteoclastogenesis but increased trabecular bone^16^. The exact reason why the osteoporotic phenotype was accompanied paradoxically with decreased OC differentiation and function in vitro is not clear. We assumed that these conflicting results may be due to an attempt to compensate for the increased RANKL expression in the bone microenvironment as a result of PKC-δ cKO in B cells. We demonstrated that RANKL was significantly increased both in the serum and in the bone sections in vivo after PKC-δ ablation. However, when the BMMs were isolated for osteoclastogenesis in vitro and an equal amount of RANKL was applied, it is possible that BMMs from PKC-δ cKO mice, which were pre-exposed to high concentration of RANKL in vivo, became less responsive when stimulated with RANKL in vitro.

Importantly, the positive role that RANKL has in activating the immune system appears to significantly contribute to pathologic bone loss^40^. Furthermore, RANKL has also been demonstrated to play essential roles in lymphocyte development and lymph node organogenesis^41,42^. These observations have spurred intense study of the various ways in which the immune system can influence bone^43^. RANKL exists in two forms: a membrane-bound form and a soluble form, and both forms were shown to be biologically capable of enhancing OC differentiation^44^. RANKL-RANK signaling also regulates OB differentiation^27,45,46^. Surface-bound RANKL expressed on OB transferred reverse signals from the exterior of the cell to the interior, which regulated OBs^47^. Moreover, osteoblastic RANKL functions as a coupling signal acceptor that recognizes vesicular RANK^48^. However, we found that soluble RANKL, unlike membrane-bound RANKL, had no effect on osteoblast formation and function. In our study, we demonstrated that both forms of RANKL were remarkably elevated in mice with PKC-δ deficiency in B cells by ELISA assay and IHC staining. Moreover, we demonstrated that PKC-δ deficiency in B cells was accompanied by changes in B-cell number, function, and distribution, especially in the spleen. These results of our study are consistent with previous findings which reported that PKC-δ negatively regulated B-cell proliferation and played a pivotal role in establishing and controlling antigen-induced B-cell tolerance^9,10^, further attesting that PKC-δ signaling plays an important role in B-cell development and RANKL production in B cells.

It is interesting that changes in the bone phenotype and B-cell numbers were more pronounced in male mice than in female mice after PKC-δ ablation in this study. Gender differences are poorly understood and are not well described in the hormone-related-gene deficient mice, such as aromatase-deficient mice^49^ and estrogen receptor knockout mice^50^. However, given that androgen receptor (AR) function is indispensable for male bone formation and remodeling^51,52^, and that androgens regulate PKC-δ transcription and modulate its apoptotic function in prostate cancer cells^53^, and prenatal testosterone exposure induces hypertension in adult females via an AR-dependent PKC-δ-mediated mechanism^54^, it is perhaps not surprising that gender differences were observed in this study. These studies suggest that PKC-δ has interactions with the AR and lead us to speculate that PKC-δ may regulate bone mass in a sex dependent manner through AR. However, the precise underlying molecular mechanisms regarding sexual dimorphism need to be further investigated.

**Fig. 7:**
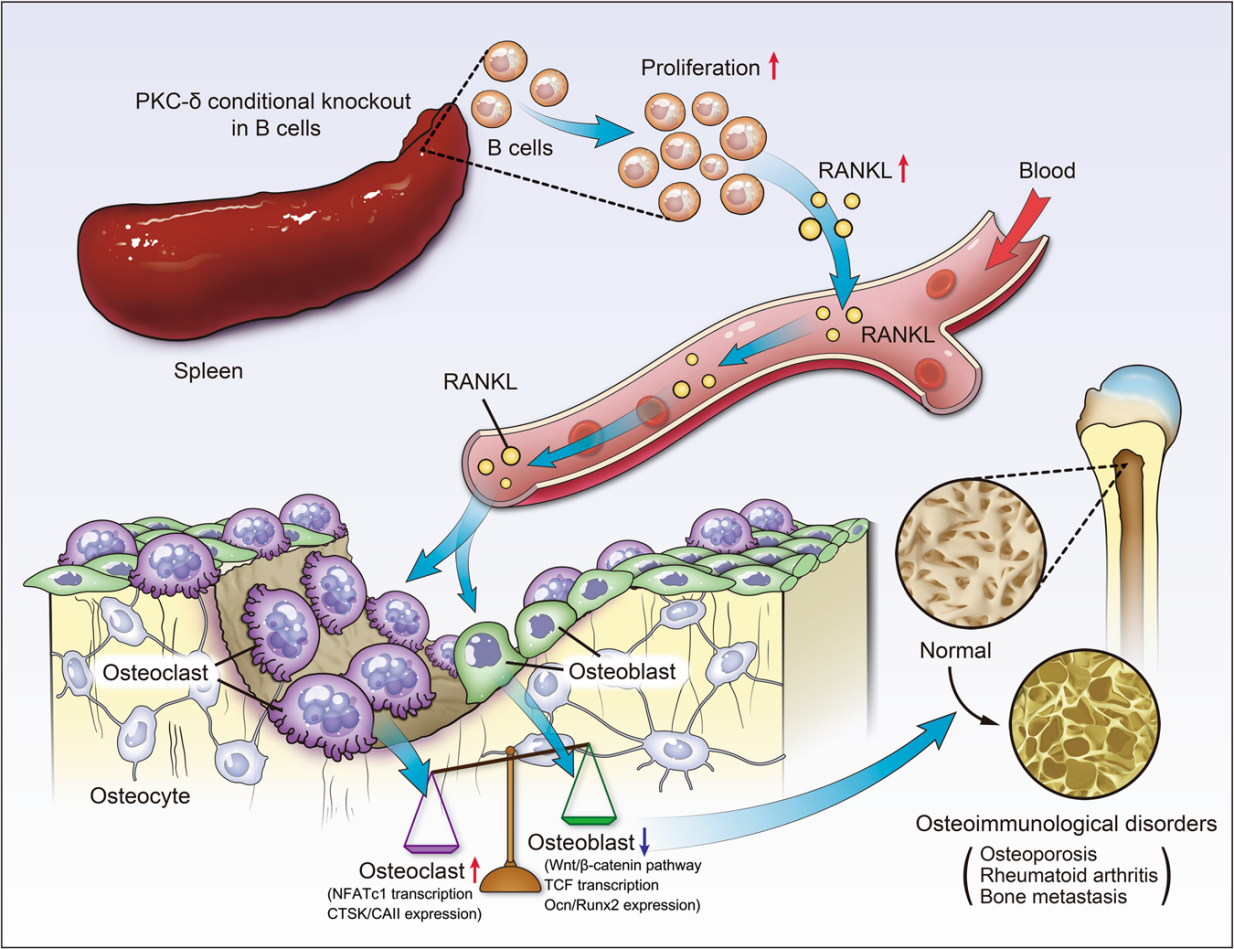
The schematic model of the hypothesized mechanism by which PKC-δ ablation selectively in B cells affects bone remodeling. PKC-δ deficiency in B cells favors bone mass loss, which is owing to the overexpression and secretion of RANKL in these B cells of hyperproliferation and the subsequent osteoclast–osteoblast uncoupling.

In summary, our data clearly demonstrate that mice with PKC-δ deficiency in B cells favored bone mass loss, which is accompanied by the overexpression and secretion of RANKL in these B cells of hyperproliferation, as well as OC–OB uncoupling (schematic model of the hypothesized mechanism was illustrated in Fig. 7). These findings are of great significance for providing novel insights into the tight coupling between PKC-δ deficient B cells and bone cells. Detailed molecular understanding of the role of PKC-δ in both the immune and skeletal systems, as well as providing a molecular basis for developing innovative strategies and therapeutic agents against osteoimmunological disorders, will surely benefit both clinical and basic research in broader disciplines.

## Materials and methods

### Animal procedures and animal care

All transgenic mice had a C57BL/6 J and C57BL/6 N genetic background. Mice were group-housed under conventional conditions: 12-h light/dark cycle, standard rodent chow and water *ad libitum* in standard cages. All mice were maintained at National Resource Center for Mutant Mice Model Animal Research Center of Nanjing University in China according to institutional guidelines. All the experiments were approved by the Institutional Animal Care and Use Committee of The Third Affiliated Hospital of Sun Yat-sen University (Approval No: IACUC-F3-18-1202) and were performed according to EU Directive 2010/63/EU.

### Generation of B-cell-specific PKC-δ conditional knockout mice

LoxP mice were purchased from RIKEN BioResource Research Center (Stock Number: RBRC06462, Strain Name: C57BL/6-Prkcd<tm1Shb>, 3-1-1 Koyadai, Tsukuba, Ibaraki 305-0074, Japan), and CD19-Cre mice were purchased from The Jackson Laboratory (Stock Number: 006785, Strain Name: B6.129P2(C)-CD19^tm1(cre)Cgn^/J, Bar Harbor, ME, USA). Mice with B-cell-selective knockout of the PKC-δ (CD19-Cre^+^ PKC-δ^flox/flox^) were generated by crossing mice heterozygous for a floxed exon 7 PKC-δ allele (PKC-δ^(ex7)flox/+^) with heterozygous mice carrying a cyclization recombinase of which the expression is controlled by the CD19 promoter (CD19-Cre^+^ PKC-δ^flox/+^). Offspring mice were weaned at 3 weeks of age and genotyped by PCR, the presence of the CD19-Cre transgene was determined on genomic DNA via PCR with forward primer: 5′-ATTTGCCTGCATTACCGGTC-3′ and reverse primer: 5′-ATCAACGTTTTCTTTTCGG-3′ resulting in a 377 bp fragment. CD19-Cre^-^PKC-δ^flox/flox^ littermate mice were used as control.

### Micro-CT measurement

Microstructure of bone in mice were measured by high-resolution micro-CT as previously described^55,56^. Micro-CT scanning was performed in vitro on the right tibia and femur using a ScancoμCT100 scanner (Brüttisellen, Zurich, Switzerland) with a fixed isotropic voxel size of 10 μm, 100 slices, 70 kV at 200 μA, 300 ms integration time. Micro-CT parameters were evaluated in the trabecular region of the distal femur and proximal tibia, commencing at a distance of 0.5 mm from the growth plate and extending a further 1.5 mm proximally for femur and distally for tibia. Parameters assessed included bone volume fraction (bone volume/total volume, BV/TV, %), trabecular number (Tb.N, mm^−1^), trabecular thickness (Tb.Th, mm) and trabecular separation (Tb.Sp, mm). Cortical bone was analyzed starting at a distance of 2.75 mm from the growth plate and extending 1 mm towards the mid shaft to determine total cortical area (Tt.Ar, mm^2^), cortical bone area (Ct.Ar, mm^2^), cortical area fraction (Ct.Ar/Tt.Ar, %), and cortical thickness (Ct.Th, μm).

### Bone histomorphometry and immunohistochemistry

Left tibias were fixed overnight in 10% buffered formalin, decalcified with 14% EDTA for 7 days, embedded in paraffin and histological sections of 3 μm were then prepared for staining. Trabecular bone parameters and in vivo OB parameters were assessed from H&E stained sections as described^57^, while in vivo OC parameters were obtained from TRAP staining as previously^58^. Trabecular bone region of interest was measured 500 µm below the growth plate and 1 mm in height at the proximal tibia. Histomorphometric analysis was performed by quantifying parameters including osteoclast surface per bone surface (Oc.S/BS), number of osteoclasts per bone perimeter (N.Oc/B.Pm), osteoblast surface per bone surface (Ob.S/BS) and number of osteoblasts per bone perimeter (N.Ob/B.Pm) using an Olympus microscope and the BIOQUANT OSTEO software (BIOQUANT OSTEO 2013 Ver.13.20.6, Nashville, TN, USA). We counted the numbers of positively stained cells in five sequential sections per mouse in each group and normalized them to the number per millimeter of adjacent bone surface or per square millimeter.

For bone tissue IHC, antigen retrieval was carried out by incubating specimens with bone tissue specific antigen-retrieval solutions (SBT100013, Showbio, Shanghai, China) for 60 min at 37 °C before commencing with IHC staining protocol. Non-specific binding was blocked with goat serum for 30 min at room temperature before incubation with primary antibody (CTSK ab19027, RANKL ab45039, OPG ab9986 and osteocalcin ab93876. Abcam, Cambridge, UK) against mouse antigens at 4 °C overnight in a humidified container. For detection, sections were treated with HRP-conjugated secondary antibody (GK500505A, Dako, Carpinteria, CA, USA) for 30 min at 37 °C, followed by DAB substrate (ZLI-9017, ZSGB-Bio, Beijing, China) for 30 seconds, counterstained with Mayer’s hematoxylin, dehydrated, and mounted. For negative controls, sections were treated with the same amount of primary antibody diluent without primary antibodies. Semi-quantitative evaluation of CTSK, RANKL and OPG was performed as previously described^59^ in five random trabecular regions (mainly OCs and OBs) of each section. Briefly, staining intensity (A) was classified as 0 (negative), 1 (weak), 2 (moderate) and 3 (strong). The percentage of positive cells (B) examined in 100 cells were divided into 0 (<5%), 1 (5–25%), 2 (26–50%), 3 (51–75%) and 4 (>75%). The IHC result (possibly 0, 1, 2, 3, 4, 6, 8, 9, 12) was the product of A and B and classified as 0 (equal to negative (−)); 1–4 (weakly positive (+)); 5–8 (moderately positive (++)) and 9–12 (strongly positive (+++)). Two experienced pathologists scored each section, they were blinded to the identity of each specimen at the time they determined the score and the final scores were evaluated by consensus.

Safranin O Fast Green Staining, Masson’s trichrome staining and Von Kossa staining (in undecalcified sections) were used to assess chondrocytes, organic, and inorganic matrix components, respectively. Von Kossa staining was performed by incubating sagittal nondecalcified sections (4 μm) of the left femur with 1% silver nitrate solution under ultraviolet light.

### Real-time RT-PCR assay

Total RNA was isolated with a total RNA extraction kit (R6834, Omega, USA) and reverse transcription was carried out using 1 μg of total RNA with reverse transcriptase (RR036, Takara Bio, Japan) in a volume of 20 μl according to the manufacturer’s instructions. One microliter of cDNA was amplified with the specific primers (Invitrogen) as follows and were quantified on a Light Cycler 480II (Roche) using multiple kits (4887352001, SYBR Green I Master, Roche), normalizing with GAPDH. PCR primers were: mouse *pkc-δ*, 5′-ACCCAGAAGACTGTGGATGG-3′ (forward) and 5′- CGTCCCTGTCTAGCATCACA-3′ (reverse); mouse *nfatc1*, 5′-CCGTTGCTTCCAGAAAATAACA-3′ (forward) and 5′- TGTGGGATGTGAACTCGGAA-3′ (reverse); mouse *ctsk*, 5′-CCAGTGGGAGCTATGGAAGA-3′ (forward) and 5′- AAGTGGTTCATGGCCAGTTC-3′ (reverse); mouse *calcitonin receptor*, 5′-TGCAGACAACTCTTGGTTGG-3′ (forward) and 5′- TCGGTTTCTTCTCCTCTGGA-3′ (reverse); mouse *ocn*, 5′-GCGCTCTGTCTCTCTGACCT-3′ (forward) and 5′-ACCTTATTGCCCTCCTGCTT-3′ (reverse); mouse *col1a1*, 5′-CTGGCGGTTCAGGTCCAAT-3′ (forward) and 5’- TCCAAACCACTGAAGCCTCG-3′ (reverse); mouse *runx2*, 5′-CGCATTCCTCATCCCAGTAT-3′ (forward) and 5′- TGTAGGTAAAGGTGGCTGGG-3′ (reverse); mouse *gapdh*, 5′-CACATTGGGGGTAGGAACAC-3′ (forward) and 5′-TGAGTCCTTCCACGATACCAAAGTT-3′ (reverse); all experiments were repeated three times. The Ct value of the reference gene GAPDH was subtracted from the Ct value of the target genes (ΔCt), and the average ΔCt value of triplicates was taken. Relative expression levels of each gene were obtained by using the 2^−ΔΔCt^ method.

### Western blotting assay

Cells were lysed in RIPA buffer containing 20 nM Tris-HCl, 150 mM NaCl, 1% Triton X-100, 0.2% deoxycholate, protease, and phosphatase inhibitors for 30 min on ice for protein extraction. Protein concentrations of cell lysates were determined by using a BCA assay. An equal amount of proteins (30 μg/lane) was resolved by SDS-polyacrylamide gel electrophoresis and then transferred to the PVDF membrane (Millipore, Boston, MA, USA). The membrane was probed with the indicated primary antibodies overnight at 4 °C. Antibodies were purchased from Abcam (PKC-δ, ab182126; CAII, ab124687), Cell Signaling Technologies (GSK-3β, #12456; phospho-GSK-3β, #5558; TCF, #2203; RUNX-2, #12556; β-catenin, #8480) and SANTA CRUZ (CTSK, sc-48353; NFATc1, sc-7294; GAPDH, sc-66163; α-Tubulin, sc-69970). Blots were then developed using HRP-conjugated secondary antibodies for 60 min at room temperature and were visualized by using enhanced ECL reagents (Amersham, Pittsburgh, PA, USA) according to manufacturer’s instructions. Images were acquired on an Imagequant LAS 4000. α-Tubulin or GAPDH was used as a loading control to ensure equal amounts of protein were loaded per lane. Experiments were repeated three times and quantification of all the blots is presented as mean ± SD. Signal intensities were quantified by NIH ImageJ software.

### B-cell isolation, purification, and culture

For primary B-cell purification, single-cell suspensions were prepared from pooled spleens of WT and PKC-δ cKO mice. Cell suspensions were depleted of red blood cells by ammonium chloride lysis buffer (containing 0.15 M NH4Cl, 10 mM Tris-HCl, 0.1 mM EDTA). B cells were purified using the B-Cell Isolation Kit (MiltenyiBiotec, 130-121-301) according to the protocol of the manufacturer. The purity of B cells was more than 95%, which was confirmed by flow-cytometric analysis using anti-CD19 antibody (BD Biosciences, Catalog # 550992).

For in vitro B-cell culture, enriched primary mouse B cells were cultured in RPMI 1640 (HyClone) containing 10% FBS, 50 nM 2-ME, 4 mM l-glutamine, and 1% penicillin/streptomycin. Cells were stimulated with 4 μg/ml CpGODN 2006 (CPG, Invivogen) and 1 μg/ml trimeric CD40L (R&D systems). Phor-bol-12-myristate-13-acetate (PMA, 50 ng/ml, Sigma), ionomycin (500 ng/ml, Sigma) and Brefeldin A (BFA, 10 mg/ml, Sigma) were added at the indicated concentrations at the last 6 h.

For preparation of B-cell supernatant for OB and OC co-cultures, mouse B cells were purified from splenocytes using the B-cell isolation kit and 2 × 10^6^ cells/ml grown in RPMI 1640 supplemented with 10% FBS, CPG and CD40L. This growth medium was collected at day 4 as B-cell supernatants. The supernatants were concentrated 10-fold through Amicon Ultra-15 centrifugal filter devices with a NMWL-3KD device (Millipore, Billerica, MA, USA) before use. For the co-culture experiments, 25 μl B-cell supernatant per milliliter complete culture medium was used as described^60^.

### Flow-cytometry assay

For phenotypic analysis, single-cell suspensions were prepared from spleen, lymph nodes, liver and thymus. Briefly, lymph nodes and thymus were passed through a 200-gauge steel mesh and washed with PBS. Spleens were firstly passed through a 200-gauge steel mesh and then RBC were lysed and remaining cells washed with PBS. Livers were passed through a 200-gauge steel mesh and the cell pellets were collected in the centrifuge tube, the mononuclear cells in the pellets were isolated by gradient centrifugation with 40% and 70% Percoll. Single-cell suspensions were stained according to the manufacturers’ instructions using fluorochrome-coupled monoclonal antibodies (mAbs). The following mAbs were obtained from BD Bioscience: BV421-conjugated anti-CD45.2 (Catalog # 562895), PE-conjugated anti-CD5 (Catalog # 553022), APC-conjugated anti-CD19 (Catalog # 550992). PE-Cy7-conjugated anti-IL-10 (Catalog # 505026) was purchased from Biolegend. Flow-cytometric analyses were performed using an LSR II flow cytometer (BD Biosciences) and flow-cytometry data were analyzed using Flowjo V10 software (BD Biosciences).

### Cryosectioning and immunofluorescent assay

Spleens were harvested, fixed with 4% paraformaldehyde, washed in PBS, and dehydrated overnight in 30% sucrose (Sigma). The samples were embedded in Tissue-TekOCT compound (Bio-Optica) and frozen in an ethanol dry ice bath. Sections of 7 μm were placed on glass slides (Bio-Optica) and blocked for 30 min with PBS-Tween 0.05% plus 0.5% FBS. Sections were stained with Abs directed against B220 (Alexa Fluor 594, Biolegend, 103254, San Diego, CA, USA) for 1 h at 37 °C to visualize B cells, nuclei were counterstained using DAPI (KGA215, Jetway, Nanjing, China) for another 15 min at 37 °C, then coverslips were mounted on a microscope slide with Antifade Mounting Medium (P0126, Beyotime, Jiangsu, China). Finally, Images were acquired using a laser scanning confocal microscopy (LSM880, Zeiss, Jena, Germany).

### ELISA assay

Mouse blood was obtained from the Fundus vein. Samples were allowed to clot for 2 h at room temperature and then centrifuged for 15 min at 12,000 rpm at 4 °C. Serum was collected and frozen at −20 °C until use. OPG (Catalog # EMTNFRSF11B) and RANKL (Catalog # EMTNFSF11) ELISA kits were purchased from Invitrogen, while β-CTX (Catalog # E-EL-M0372c) and PINP (Catalog # E-EL-M0233c) ELISA kits were obtained from Elabscience. Serum OPG, RANKL, β-CTX and PINP produced in vivo by WT and PKC-δ cKO mice were measured by ELISA assay according to the manufacturer’s instructions. All the optical densities (ODs) measured after reactions were converted to the concentration using their standard curves. All the samples were measured in triplicate.

### Macrophage isolation from mouse bone marrow, osteoclastogenesis assay, and tartrate-resistant acid phosphatase staining

Bone marrow from tibia and femur of WT and cKO mice was flushed with α-MEM and cells pelleted by centrifugation at 1500 rpm for 5 min. Bone marrow cells were then cultured in complete α-MEM media supplemented with 10% fetal bovine serum, 1% antibiotic, and 30 ng/ml of recombinant mouse macrophage colony stimulating factor (MCSF, 416-ML, R&D systems, Minneapolis, MN, USA) at 37 °C in a humidified 5% CO_2_ atmosphere to allow cell attachment. After 2 days, non-adherent cells were removed and discarded; adherent cells were cultured to confluence and treated as bone marrow macrophages (BMMs). BMMs of passage 1 or 2 were used in the experiments below. BMMs were seeded on to 96-well plate (6 × 10^3^ cells/well) and treated with 30 ng/ml MCSF, and 100/25 ng/ml RANKL (462-TEC, R&D systems) to induce OC formation. The media was replaced with supplemented MCSF and RANKL (with and without BS) every 2 days and after 7 days of culture, the cells were fixed in 2.5% glutaraldehyde and stained with Acid Phosphatase staining kit (387 A, Sigma–Aldrich, St. Louis, MO, USA) according to the protocol of the manufacturer. TRAP-positive multinucleated cells with more than three nuclei were counted as OCs using light microscopy. The number of OCs in each well was counted using ImageJ software.

### Hydroxyapatite resorption assay

The diluted BMMs cell suspension (100 μl, 5 × 10^3^ cells per well) was transferred into each well of a 96-well Corning Osteo Assay Surface plate (3989, Corning Life Sciences, Tewksbury, MA, USA) coated by hydroxyapatite to begin the differentiation process. Plates were incubated at 37 °C in a humidified atmosphere of 5% CO_2_ with differentiation medium (30 ng/ml MCSF combined 100 ng/ml RANKL) (with and without BS) changed every 3 days. After 7 days, the plates were stripped with 1.2% sodium hypochlorite solution for 5 min to remove cells, rinsed with distilled water, and air-dried prior to imaging. Overlapping images of the entire well surface were taken at ×20 magnification and these were then used to produce a composite image using Image Composite Editor (ICE 2.0, Microsoft, USA). The total resorption area was then measured in the composite image using Image-Pro Plus (version 6.0, Media Cybernetics Company, Rockville, MD, USA).

### In vitro osteoblast differentiation assay and CCK-8 measurement of osteoblast proliferation

OB precursors were obtained from calvaria of 6-week-old mice after digesting with collagenase II solutions as described previously^61^. Cells were seeded at a density of 1 × 10^6^ cells/ml in complete Dulbecco’s Modified Eagle’s Medium (DMEM) supplemented with 10% FBS, 2mM L-glutamine, 1% penicillin and streptomycin. Osteogenic media (10^−8^ M dexamethasone, 10 mM β-glycerophosphate and 50 μg/ml ascorbic acid) was used for culturing osteoblasts. To detect osteoblastogenesis short-time course signaling, 20 mM LiCl (Sigma, Lot # MKBZ4804V) was used as previously described^62^. BS co-culture and BMP-2 (novoprotein, Catalog #C012, Shanghai, China) stimulation (25 ng/ml) were added at the indicated time-points.

Passage two OBs were cultured at 2.5 × 10^3^ cells/well in a 96-well plate for 24 h. To evaluate the effect of PKC-δ cKO on OB viability, proliferation was measured at 24 and 48 h with the Cell Counting Kit-8 (CCK-8) kit (Dojindo Molecular Technologies, Inc., Kumamoto, Japan) after culture according to the manufacturer’s protocol. At the end of culture, 10 μl of CKK-8 was added to each well, cells were cultured for an additional 1 h, absorbance was measured at 450 nm using a micro plate reader (BioTek ELx800, BioTek Inc., Winooski, VT, USA). The experiments were performed in triplicate.

### Alkaline phosphatase staining, Alizarin red staining, and mineralization

ALP was considered as an early differentiation marker of OBs while mineralization activity occurred in the later stage. ALP staining kit (BCIP/NBT Liquid Substrate System, Catalog # B1911, Sigma) was used to test the activity of ALP on days 7 in the 48-well plates according to the manufacturer’s protocols. Alizarin red staining kit (A5533, Sigma) was used to test the mineralization function of OBs on day 21 in 24-well plates. The ALP staining intensity and the mineralized area were visualized using ELISPOT analyzer (S6 ULTRA, C. T. L, USA) and measured by ImageJ software.

### Statistical analyses

All data presented in this study are representative of one of three independent experiments, and the results are presented as mean ± SD. Statistical analyses were performed with the two-tailed Student’s *t*-test for two-group comparisons, or one-way ANOVA and post-hoc multiple comparisons with Bonfer-roni correction for three- or four-group comparisons. All data analysis was performed with SPSS 20.0 Package (SPSS software 20.0; SPSS, Chicago, IL, USA). All statistical tests were two-sided and values of *p* smaller than 0.05 were considered significant.

## Supplementary information

Supplementary Figure Legends

Supplementary Figure 1

Supplementary Figure 2

Supplementary Figure 3

Supplementary Figure 4

Supplementary Figure 5
