# Supplementary material for: PKC-δ deficiency in B cells displays osteopenia accompanied with upregulation of RANKL expression and osteoclast–osteoblast uncoupling
Source: Cell Death Dis. 2020 Sep 16;11(9):762. doi: 10.1038/s41419-020-02947-3 (PMC7494897; doi:10.1038/s41419-020-02947-3)
Supplement: Supplementary file 1 — Supplementary Figure Legends [file 41419_2020_2947_MOESM1_ESM.docx]

**Supplementary Figure Legends**

**Supplementary Fig. 1 Generation and validation of mice lacking PKC-δ in B cells.** (A) Strategy for inserting flox sites into the mouse PKC-δ gene and CD19-Cre mediated conditional knockout of PKC-δ in B cells; (B) PCR analysis of extracted DNA from tails derived from wide type (WT), PKC-δ conditional knockout (cKO) and flox control mice. The 377 bp PCR fragment is detected in PKC-δ cKO mice, indicating Cre-mediated excision; (C) Quantitative real-time PCR analysis of PKC-δ mRNA and validation of PKC-δ-targeted mouse B cell cKO in spleen-derived B cell cultures of PKC-δ cKO and WT control mice. Values are expressed as the mean of three independent experiments (n = 3 mice/group in each experiment)±SD; (D) Western Blotting analysis of PKC-δ in spleen-derived B cell cultures in PKC-δ cKO and WT control mice, validating efficacy of PKC-δ ablation for floxed allele compared to PKC-δ germline WT controls.

**Supplementary Fig. 2 Osteocalcin immunohistochemistry staining in trabecular bone of tibia revealed decreased osteoblast after PKC-δ ablation in B cells.** (A) Representative images of osteocalcin immunohistochemistry staining sections form PKC-δ cKO and WT control mice. Osteoblasts on bone surface were discerned by morphology and calculated by two independent observers blinded to the groups. (B) Number of osteocalcin positive (N.Ocn^+^) on the bone surface was measured as cells per millimeter of perimeter in sections (/B.Pm). (C) The mean density of the corresponding positive cells was calculated as integrated optical density per area of positive cells by detecting in 6 different images taken at 100x magnification with Image Pro Plus 6.0 software. Six mice per group were examined. Three equidistant sections spaced at 200μm apart throughout the mid-sagittal section of tibia were evaluated. Yellow arrows in A indicated positive osteoblasts. Bar represents 100μm. * *p*<0.05 compared with WT control group.

**Supplementary Fig. 3 Rrepresentative images of bone component analysis of 12-week-old wide type and PKC-δ conditional knockout mice.** (A) Representative images of safranin O staining from proximal tibia to determine articular cartilage; (B) Representative images of Masson staining from proximal tibia to visualize changes of organic elements in bone; (C) Representative images of von Kossa staining from distal femur to determine changes of inorganic elements in bone. Higher magnification micrographs of area within square in A–B were presented at the right side of each. Arrows in C indicate the trabecular bone. Red bars and black bars represent 200μm and 100μm respectively.

**Supplementary Fig. 4 The gating strategy of CD19^+^CD5^+^ B1a cells.** The dot diagram shows the gating process of B1a cells, CD19^+^CD5^+^ cells are defined as B1a cells.

**Supplementary Fig. 5 Exogenous RANKL has no osteogenic effect on MC3T3-E1**. (A) MC3T3-E1 cells were seeded in a 48-well plate at a density of 1×10^4^ cells/cm^2^ and maintained in α-MEM supplemented with 10% FBS, 1% Pen/Strep and 1% glutamax at 37ºC in a humidified incubator with 5% CO2. MC3T3-E1 cell differentiation was induced by osteogenic differentiation medium containing 2mM β-glycerophosphate and 50μg/ml ascorbic acid with or without 50ng/ml BMP-2. Culture media containing 50ng/ml and 100ng/ml soluble RANKL was changed every second day. Cells were fixed in 2.5% glutaraldehyde at day 7 and were then stained for ALP (BCIP/NBT liquid substrate system) for 15-20min before rinsing with ddH2O. (B) Semi-quantification was analyzed by ImageJ software (NIH). * *p*<0.05 compared with Negative Control Group without BMP-2 and RANKL stimulation.
