# Supplementary figures and images for: PKC-δ deficiency in B cells displays osteopenia accompanied with upregulation of RANKL expression and osteoclast–osteoblast uncoupling
Source: Cell Death Dis. 2020 Sep 16;11(9):762. doi: 10.1038/s41419-020-02947-3 (PMC7494897; doi:10.1038/s41419-020-02947-3)

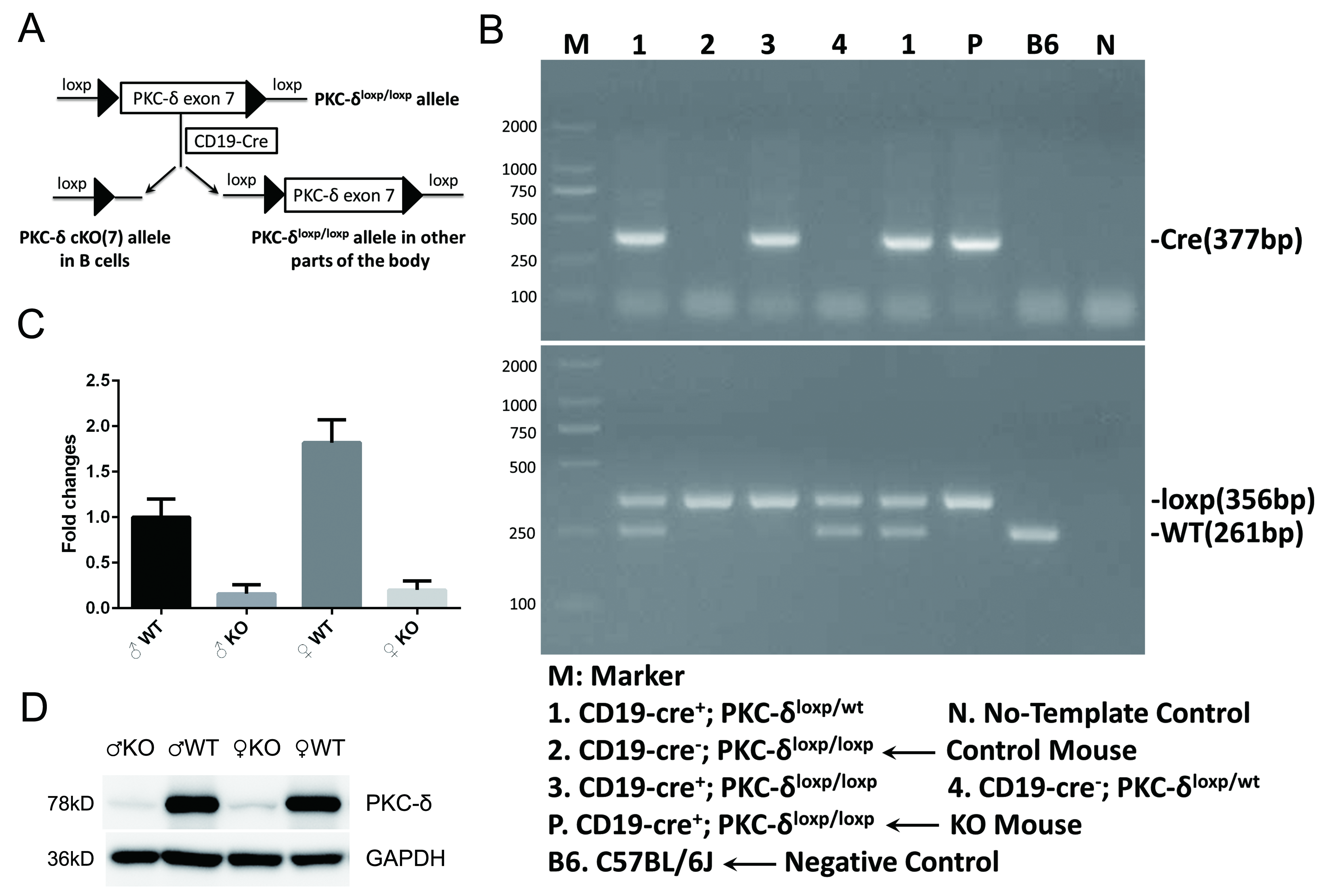

Supplement: Supplementary file 2 — Supplementary Figure 1 [file 41419_2020_2947_MOESM2_ESM.tif]

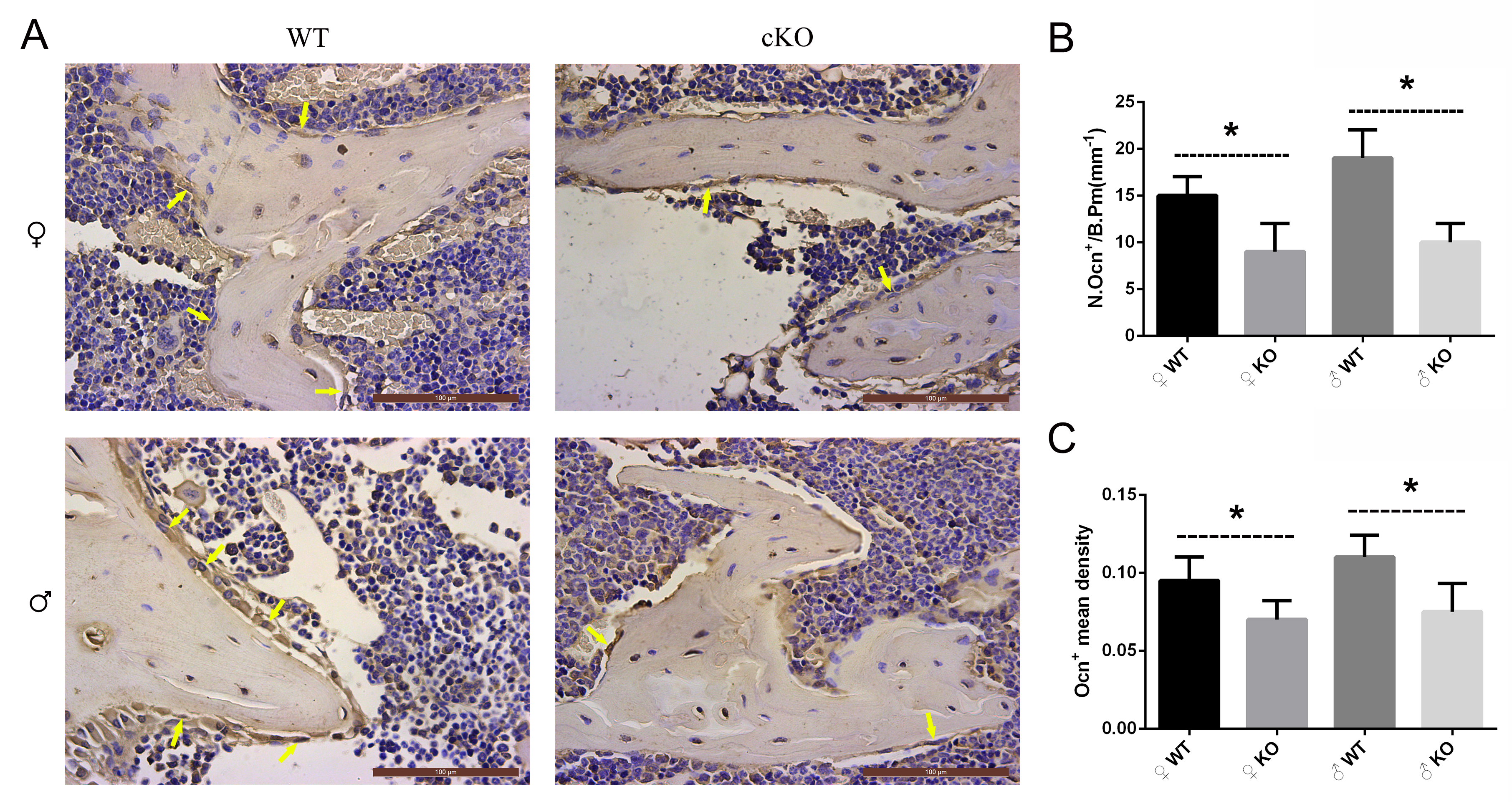

Supplement: Supplementary file 3 — Supplementary Figure 2 [file 41419_2020_2947_MOESM3_ESM.tif]

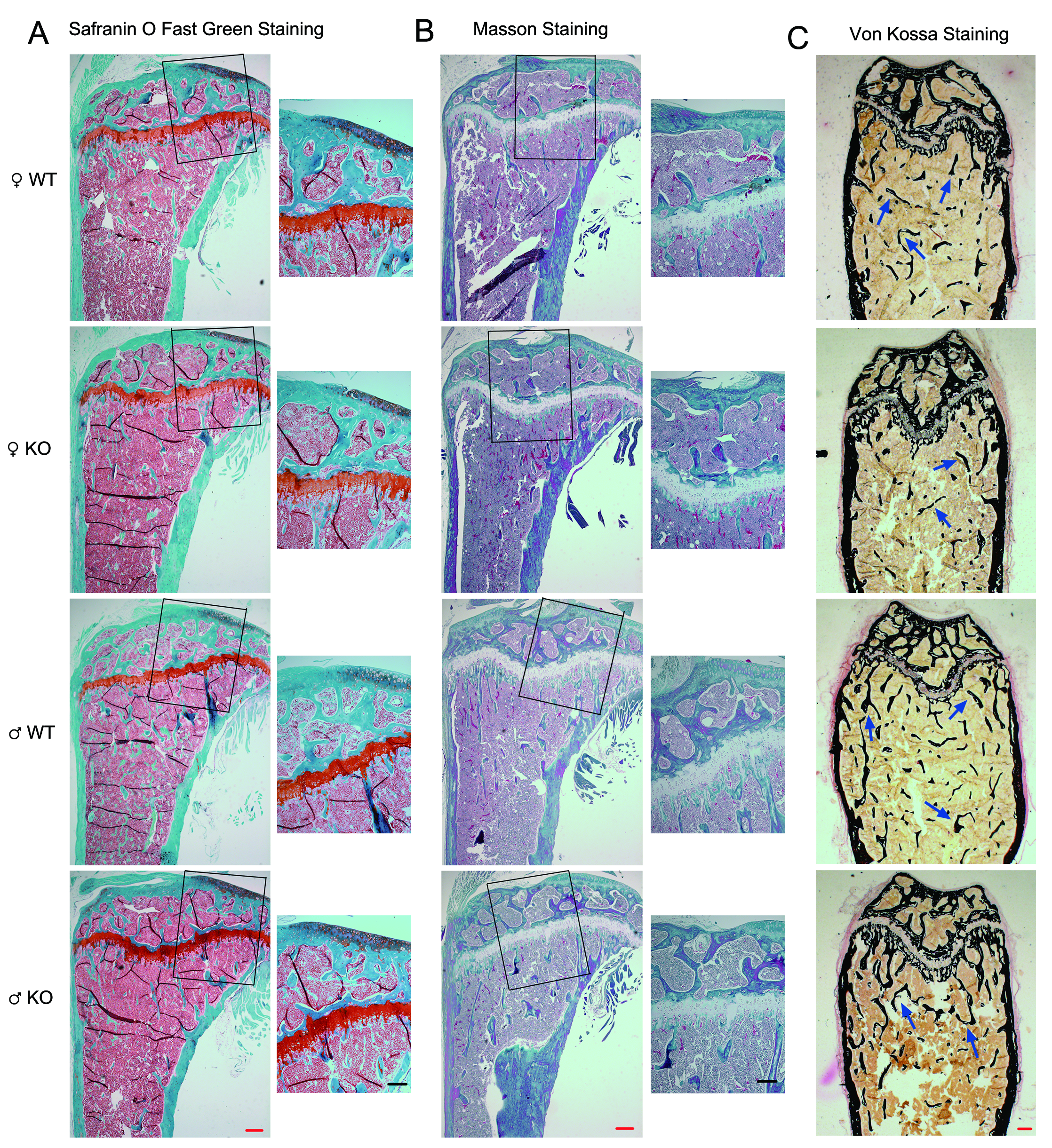

Supplement: Supplementary file 4 — Supplementary Figure 3 [file 41419_2020_2947_MOESM4_ESM.tif]

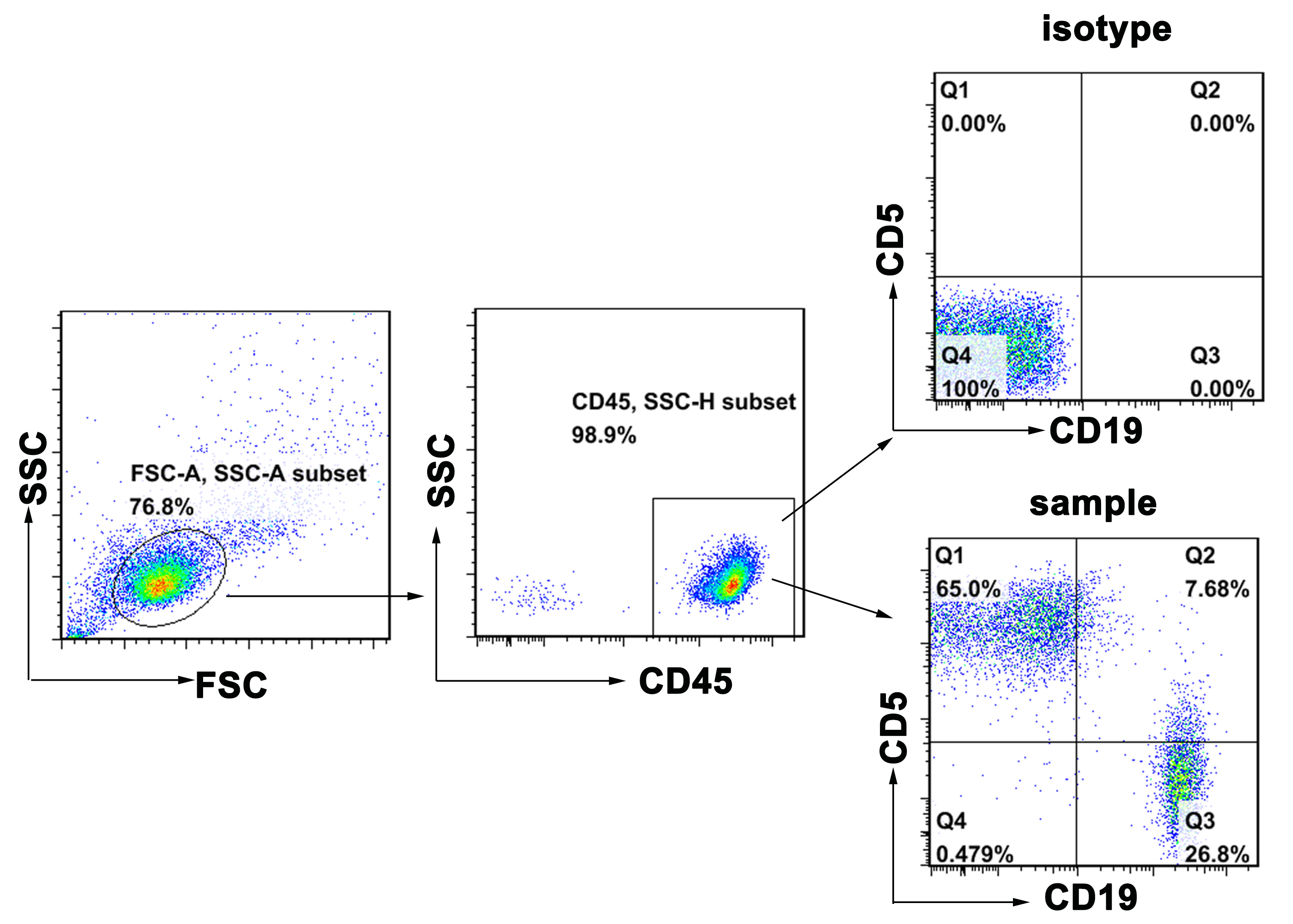

Supplement: Supplementary file 5 — Supplementary Figure 4 [file 41419_2020_2947_MOESM5_ESM.tif]

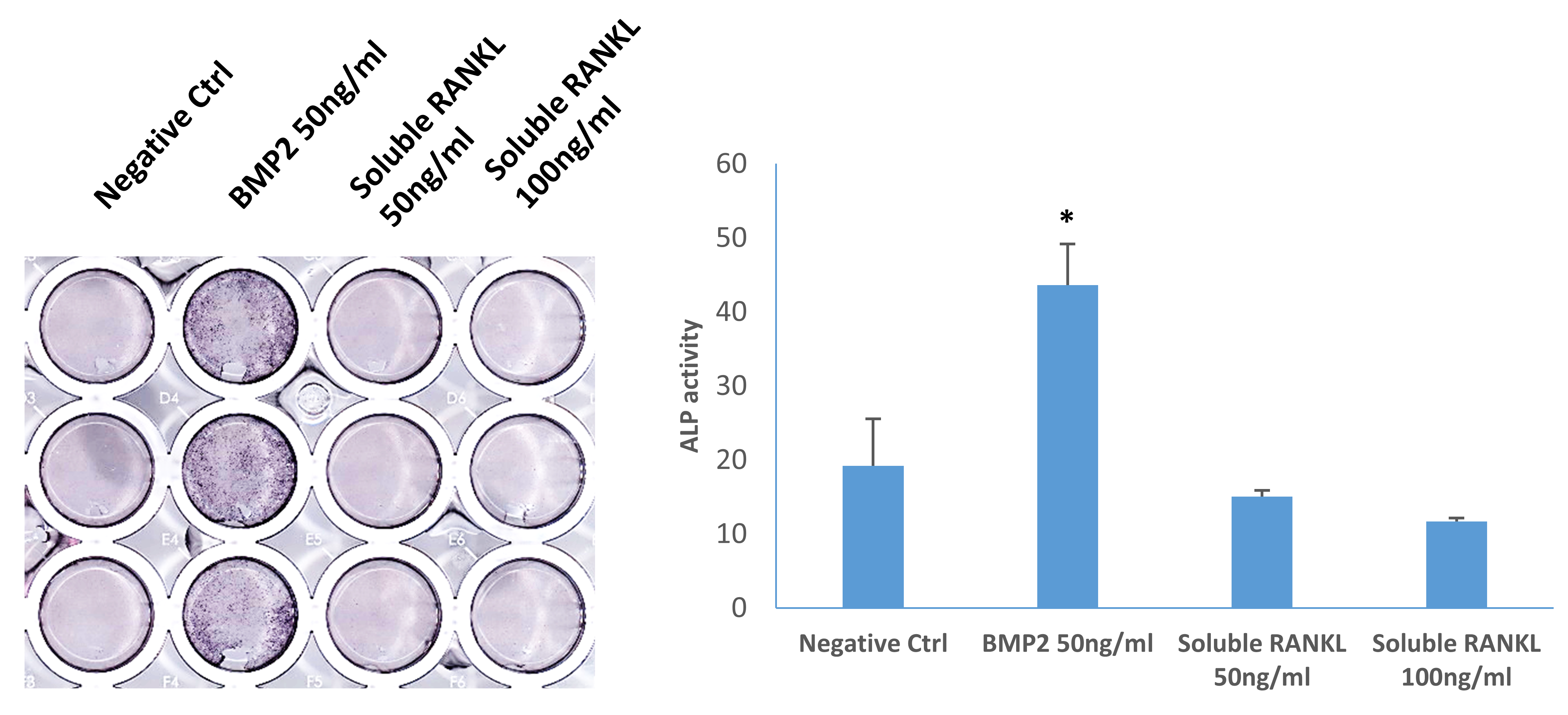

Supplement: Supplementary file 6 — Supplementary Figure 5 [file 41419_2020_2947_MOESM6_ESM.tif]
